# First-time serological and molecular detection of *Helicobacter pylori* in milk from Algerian local-breed cows

**DOI:** 10.14202/vetworld.2018.1326-1330

**Published:** 2018-09-25

**Authors:** Meryem Guessoum, Zehor Guechi, Mounir Adnane

**Affiliations:** 1 Department of Pre-Clinical Medicine, High National Veterinary School, BP161 El-Harrach, Algiers, Algeria; 2 Department of Microbiology, Central Laboratory of Clinical Biology, University Hospital Center of Nafisa Hamoud (Parnet), Hussein Dey, Algiers, Algeria; 3 Department of Clinical Medicine, Institute of Veterinary Sciences, Tiaret, Algeria

**Keywords:** Algeria, cows, feces, *Helicobacter pylori*, milk, serum

## Abstract

**Aim:**

The present study was conducted to detect and identify *Helicobacter pylori* within local cow breeds in the central region of Algeria.

**Materials and Methods:**

Two hundred (n=200) cows from three provinces of the central region of Algeria were studied, between January 2016 and September 2017. Each cow was subject to stool, milk, and blood sampling. Milk and fecal samples were used to detect and identify *H. pylori* using bacteriology culture method. Blood and milk samples were used to detect *H. pylori* immunoglobulin G (IgG) antibody using enzyme-linked immunosorbent assay. Polymerase chain reaction was used to confirm the abundance of *H. pylori* in milk by detecting *glmM* gene.

**Results:**

Out of 200 sera and 200 milk samples, 12% (24) and 4% (8/200) were positive for the *H. pylori* IgG antibody. *glmM* gene was detected in the milk of 13% of cows and was confirmed in all cows presenting IgG in milk.

**Conclusion:**

From the present study, we concluded that the *glmM* gene is an important marker for detecting *H. pylori* in milk. Moreover, Algerian local-breed cows are a source of *H. pylori* and could be responsible for serious zoonosis.

## Introduction

*Helicobacter pylori* is a Gram-negative, microaerophilic bacterium. It has been detected in half of the human population [1-4]. Prevalence of *H. pylori* infection varies widely according to a geographic area, age, race, and ethnicity [5]. Developed countries are more exposed to *H. pylori* infection, as hygiene of animals, material, and personal is difficult to control in large herds, which would increase dramatically the transmission of the bacteria to humans [6]. Surprisingly, *H. pylori* infection seems to be in continuous increase in developing countries [7-9]. This increase might result from the efforts of increasing the production of animal originated food to respond to the increased population, without ensuring the quality of the production such as the hygiene if staff and herd.

*H. pylori* has a strong affinity to stomach mucosa and induce serious diseases in the gastrointestinal tract such as chronic gastritis, duodenal ulcer, and gastric cancer [10,11]. For that, the World Health Organization considers *H. pylori* as an important Class I carcinogen factor [11]. Even though humans are the principal reservoir of *H. pylori* [12,13], it could spread through food and water by fecal-oral and oral-oral routes and colonize the stomach and intestines of humans and several animal species [12,14].

Professionals dealing with animals and animal-originated food such as veterinarians, butchers, and slaughterhouse staff showed high levels of antibodies against *H. pylori* [15,16] suggesting that ruminants might be a source of contamination for humans. *H. pylori* was isolated from milk of different farming animals mainly cow, ewe, camel, and sow [17,18]. These findings confirm that farming animals are a potential source of *H. pylori* and represent a risk of contamination for humans handling them or consuming their originated products such as meat and milk.

As cow milk represents the most consumed milk in the world, we think that it may represent a potential source of human contamination by *H. pylori*. However, in Algeria milk is consumed mainly as raw milk or transferred to dairy products and its consumption is higher in the central part of Algeria which is known as the principal region of dairy farming. Therefore, we think that milk originated from cows in this region could be one of the main sources of transmission of *H. pylori* to humans. Our hypothesis is supported by the high prevalence of *H. pylori* infection in humans reported in different studies in the central region of Algeria [19,20].

*H. pylori* infection induces chronic inflammation leading to the development of immunoglobulin G (IgG) [21], which could be measured in blood easily and with high specificity of 90% [22]. Detecting pathogenic factors of *H. pylori* can be done by polymerase chain reaction (PCR) and *glmM* gene which encodes for a phosphoglucosamine mutase is often targeted [23,24].

According to our knowledge, there is no available data about studies that have been done in Algeria regarding the zoonotic aspect of *H. pylori*. Therefore, the main aim of the present study is to demonstrate whether *H. pylori* is abundant in local cow breeds and their milk.

## Materials and Methods

### Ethical approval

The experimental protocol was approved by the scientific council of the High National Veterinary School of Algiers, Algeria, 2012.

### Sampling

The present study was conducted from January 2016 to September 2017 in the central region of Algeria. The existence of *H. pylori* was investigated in feces, serum, and fresh raw milk of 200 cows of the local Algerian breed. The selected cows come from six different regions located in three provinces of the central region of Algeria: Tizi-Ouzou, Bouira, and Boumerdes. Cows were selected according to lactation and age (at least 2 years old). All cows were under mixed indoor/outdoor housing condition and receiving the same diet composed of concentrate and forage.

#### Feces sampling

A small piece of stool (~5 mm in diameter; ~150 mg) was collected transrectally using a gloved hand and transferred into plastic tubes containing 1 ml of transport media (Stuart’s Transport Medium: Oxoid, France) and mixed thoroughly.

#### Blood sampling

5 ml of blood were collected from a coccygeal vein in Vacutainer tubes and let to clot. Serum was then transferred into sterile tubes after centrifugation. Serum samples were transported in the icebox and stored at 2-8°C for up to 3 days prior analysis. For *H. pylori* IgG antibodies detection, serum samples were stored at −80°C until further analysis.

#### Milk sampling

10 ml of fresh raw milk were collected into sterile sampling containers. Milk samples were filtered and stored at −20°C until further analysis, according to Bewley *et al*. [25].

### Isolation of *H. pylori*

100 µl of filtered milk and feces transport media were cultured in *Brucella* agar plates containing 7% defibrinated horse blood (Pasteur Institute, Algiers) and Skirrow’s supplement (vancomycin, 0.01%, trimethoprim, 0.05%, and polymyxin B, 2.5 IU/ml) [23] (Cat. No. SR0069, Oxoid France). Plates were incubated at 37°C with 95-99% relative humidity under microaerophilic conditions (5% O_2_, 15% CO_2_, and 80% N_2_) (Cat. No. AGOO25A, bioMérieux, France). Humidity was obtained by placing a wet paper towel in the anaerobe jars. Plates were incubated, and the growth rate was observed daily for 5-7 days. *Helicobacter* spp. was identified either as small (1 mm or less in diameter), clear, dome-shaped colonies or as a fine, and translucent lawn. Suspected colonies were confirmed for the presence of *H. pylori* on the basis of colony morphology and positive biochemical reactions for catalase, urease, and oxidase tests.

### Serological analysis

*H. pylori* IgG test was performed according to Safaei *et al*. [26]. Briefly, an enzyme-linked immunosorbent assay (ELISA) kit (*H. pylori* antibody [IgG] ELISA Kit; ABIN992624, GmbH Germany) was used and the results read by spectrophotometer at 450 nm. Reading <0.140 was considered negative; between 0.140 and 0.159 were considered equivocal, and ≥0.160 were considered positive. The equivocal results, according to the manufacturer’s instructions, must be repeated.

### PCR analysis on milk

1 ml of each milk sample was used for extraction of cDNA by a DNA isolation kit (Cat. No. ABIN412492, Roche, Germany) according to the manufacturer’s instruction, with slight modification according to Quaglia *et al*. [18]. Extracted DNA was amplified according to Rahimi and Kheirabadi [20] for the *glmM* gene of 294 bp using 5-GAATAAGCTTTTAGGGGTGTTAGGGG-3 as primer forward and 5-GCTTACTTTCTAACACTAACGCGC-3 as primer reverse. PCR reactions were performed in a final volume of 50 μl containing 25 μl Green Master Mix (Sigma), 10 μl genomic DNA as a template, 13 μl free ionized water, and 1 μl of each primer. PCR was performed using a thermal cycler (Bio-Rad, France) under the following conditions: Initial denaturation for 10 min at 94°C, 35 cycles for 1 min at 94°C, 1 min at 55°C, 1 min at 72°C, and a final extension at 72°C for 10 min.

The PCR products were electrophoresed through a 1.5% agarose gels (Bio-Rad, France) containing ethidium bromide.

A DNA ladder (Bio-Rad, France) used to detect the molecular weight of observed bands under a UV lamp. Samples inoculated with *H. pylori* were used as positive controls, and sterile distilled water was used as negative control.

### Statistical analysis

Comparisons of differences and similarities were conducted by the Chi-square test using SPSS software. Differences were considered significant where p<0.05.

## Results

Using traditional bacteriology culture technique, no *H. pylori* strains were isolated from feces and milk. Based on the ELISA test, 12% (24/200) of cows were positive for the *H. pylori* IgG antibody (Table-1).

**Table-1:** Occurrence of *Helicobacter pylori* among cows’ serum and milk samples using ELISA.

Samples	Serum			Milk
		
Locality designation	Total number	Number of positive (%)	Total number	Number of positive (%)
Bouira1	24	8 (33.3)	24	4 (16.7)
Bouira2	32	0 (0.0)	32	0 (0.0)
Bouira3	44	4 (9.1)	44	0 (0.0)
Tizi-Ouzou4	36	2 (5.5)	36	0 (0.0)
Tizi-Ouzou5	32	6 (18.7)	32	2 (6.25)
Boumerdes6	32	4 (12.5)	32	2 (6.25)
Total	200	24 (12.0)	200	8 (4.0)

*ELISA=*Enzyme-linked immunosorbent assay

**Figure-1:**
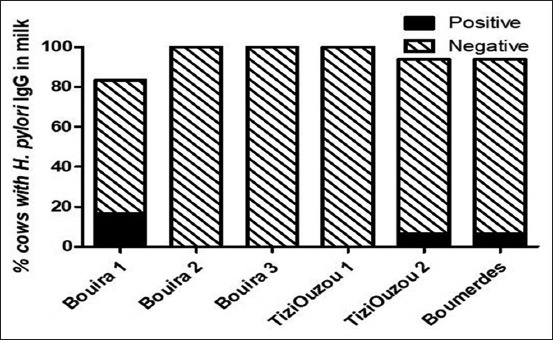
Occurrence of *Helicobacter pylori* among cows’ milk samples using enzyme-linked immunosorbent assay by region.

The results of the detection of *H. pylori* IgG revealed that out of the 200 milk samples specimens, 4% (8/200) of cows were positive (Table-1). Among the 6 studied herds, 3 had 16.7% of *H. pylori* abundance each while the other 3 herds were healthy. No statistical difference was identified between the level of contamination of different regions (Figure-1) (p>0.05).

**Figure-2:**
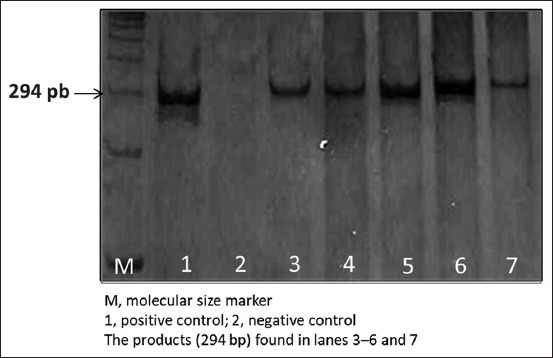
Results of the gel electrophoresis for polymerase chain reaction products of the *glmM* gene of the *Helicobacter pylori* isolates from cow milk.

**Figure-3:**
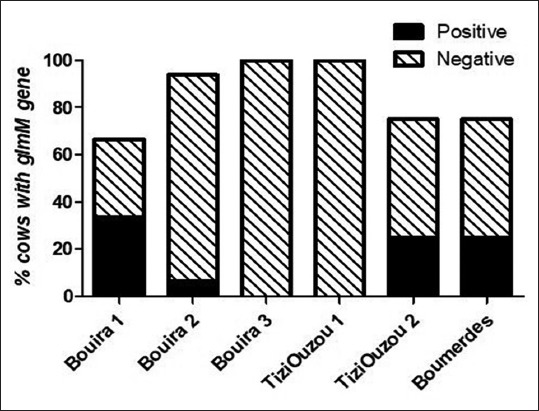
Occurrence of *Helicobacter pylori* in cows’ milk using polymerase chain reaction detection of the *glmM* gene.

The results of the detection of *H. pylori* by PCR (Figure-2) revealed that 13% of the examined cow’s raw milk was positive for the presence of *glmM* gene (Figure-3). We found that all milk samples that were positive for *H. pylori* antibody were positive for the presence of the *glmM* gene.

## Discussion

*Helicobacter* is considered emerging bacteria with major public implications [27,28]; however, its epidemiology is not fully understood [29]. In the present study, we evaluated whether cows of the local breed represent a reservoir for *H. pylori*. Different studies have been conducted in many countries, reporting the prevalence of *H. pylori* in animals with different results according to animal species and environmental factors [30,31]. This study is the first experimental work that confirms the presence of *H. pylori* in cows from different provinces of the central region of Algeria.

Determining *H. pylori* seroprevalence in cows is one of the best routine techniques to study the epidemiological aspect of the disease and the risk of human infection by these animals or their products [21,22]. As more sophisticated techniques, detecting *glmM* gene encoding for a phosphoglucosamine mutase using PCR is a potential tool for the direct detection of *H. pylori* or the confirmation of seropositive findings [24,32]. However, bacteriology is a widely used technique in the diagnosis of *H. pylori* as it gives additional information about antimicrobial susceptibility on the specific isolated strain [22].

In the present study, traditional bacteriology culture technique failed to identify *H. pylori* in raw milk and feces, leading to think that this method is not the best technique to detect *H. pylori* in animal originated products or as clinical diagnostic tool in live animals. Likewise, Tabatabaei [33] and Azevedo *et al*. [34] failed to isolate *H. pylori* from raw milk and feces of cows by classical bacteriology culture. Even in some studies where *H. pylori* were isolated, its bacteriological culture was very low [35] as only a few *Helicobacter* derived from feces of animals could be cultured according to the routine technique [36]. Furthermore, *H. pylori* can transform into coccoid form, a resistance form that makes it viable but uncultivable [11,34]. Therefore, using advanced technologies to detect the coccoid form is the best method for the study of *H. pylori* in animal-originated products.

ELISA assays revealed that 12% of the serum samples and 6% of milk samples showing the presence of IgG against *H. pylori*. The presence of IgG against *H. pylori* in bovine serum has been reported in several previous studies in different parts of the world [20,26]. In a study carried out in Iran, Safaei *et al*. [26] found that 27% of serum samples from cows were positive for *H. pylori* IgG. Likewise, Elhariri *et al*. [37] reported that 30% of cows were seropositive for *H. pylori*, using different ELISA kit.

To the best of our knowledge, there are only a few studies that have been done on animal originated food, particularly raw milk, as a source of contamination for humans by *H. pylori* [15,26]. Interestingly, we found that frequency of isolation of *H. pylori* from cows and their milk was different among the studied dairy herds and regions. Likewise, El-Gohary *et al*. [38] found a variation in the rate of contamination by *H. pylori* between different regions in Egypt. This statement could be explained by the different hygiene conditions that are mainly affected by the local culture and tradition.

In this work, we specifically targeted the *glmM* gene for molecular detection of *H. pylori* in raw milk. *GlmM* is implicated in the growth of *H. pylori* and is highly specific for the detection of *H. pylori* [39]. During this investigation, the *glmM* gene was detected in the raw milk of 13% cows. Our findings are similar to those recounted by Rahimi and Kheirabad [20], who reported that *H. pylori glmM* gene was detected in 14.1% of cow’s milk, in Iran. Interestingly, *H. pylori* was highly abundant in cow’s milk from countries with high milk production such as Japan, 72.2% [40] and Italy, 50% [18].

We think that the presence of *H. pylori* in cow’s milk is highly correlated with hygiene condition in dairy farms and the stress on the animals which is due to high milk production. Hygiene conditions are more difficult to be monitored and controlled in large herd size, as there are many materials and personally implicated in the work. Furthermore, cows with high milk yield are subject to strong stressful metabolism leading to decreasing the efficiency of the immune system [41] and systemic abundance and multiplication of *H. pylori* in cows and their milk.

## Conclusion

This study shows that *glmM* can be successfully detected in raw milk using PCR, indicating that raw milk from local breed cows in the central region of Algeria could be a potential source of zoonosis by *H. pylori*. While IgG detection in blood and milk is effective techniques for the epidemiological study of *H. pylori* in large herds, bacteriology culture is not convenient for monitoring *H. pylori* abundance in dairy cows and their originated products.

## Authors’ Contributions

MG conceived, designed the study and drafted the manuscript under the supervision of ZG. MG and MA designed the experiment protocol under the supervision of ZG. MG collected and analyzed samples. MG and MA revised the manuscript under the supervision of ZG. All authors read and approved the final manuscript.
